# Potential association between working memory and physical fitness status: the BRAVE study

**DOI:** 10.1093/eurpub/ckac131.450

**Published:** 2022-10-25

**Authors:** A Masini, F Sanmarchi, M Ricci, G Longo, ER De Gioia, A Zannoner, A Tessari, A Ceciliani, L Dallolio

**Affiliations:** 1 Department of Biomedical and Neuromotor Sciences, Alma Mater Studiorum - Università di Bologna, Bologna, Italy; 2 Department of Psychology “Renzo Canestrari”, Alma Mater Studiorum - Università di Bologna, Bologna, Italy; 3 Department of Life Quality Studies, Alma Mater Studiorum - Università di Bologna, Rimini, Italy

## Abstract

**Introduction:**

The World Health Organization launched a global action plan targeted to obtain a 15% relative reduction in the global prevalence of physical inactivity in adolescents by 2030 also promoting school-based PA interventions and programs in school. Active Breaks (ABs) are a school-based intervention consisting of short bursts (5-15 minutes) of Pa led by teachers or peer. Many researches investigated the implementation of ABs into primary school setting as a strategy to reduce sedentary behaviour, improve cognitive and physical function. However, this kind of intervention has not extended to secondary school, especially in Italian context. For this reason we started the BRAVE study to evaluate the potential effect of implementing ABs in secondary school. The study is currently in the administration phase of ABs to adolescents The preliminary analysis aims to underline a potential association between working memory performance (WM) and physical fitness status among secondary school students at baseline.

**Methods:**

In March 2022 we conducted baseline assessment in a secondary school in Valsamoggia (Bologna, Italy). Working memory was evaluated using backward digit span while physical fitness status was assessed using three different fitness test: standing long-jump (SLJ), six minute Cooper Test (6MCT) and Shuttle run test (SR).

**Results:**

A total of n = 125 adolescent, mean age 12.79±0.89, were enrolled in the study. After performing a regression analysis we found that WM is significantly associated only with age of student (b = 0.2, 95%CI 0.25, 0.11 p = 0.02). A trend also emerged between WM performance and SLJ but with no statistically significant differences (b = 0.160, 95%CI 0.03, 0.02, p = 0.09). The 6MCT and HT have no relevant associations with WM score.

**Conclusions:**

These preliminary results suggest that age is associated with cognitive performance but no positive association were found between WM score and physical fitness status excepted for a small trend with SLJ test.

**Key messages:**

• Age is related to WM in adolescent students.

• ABs interventions could represent a valid strategy to encourage movement, improve cognitive and physical fitness performance.

